# The effects of exosomes originating from different cell sources on the differentiation of bone marrow mesenchymal stem cells into Schwann cells

**DOI:** 10.1186/s12951-024-02450-3

**Published:** 2024-05-03

**Authors:** Xianxiang Zhang, Weiwei Zhang, Hao Sun, Hui Wang

**Affiliations:** Department of Otolaryngology and Head and Neck Surgery, Beijing Luhe Hospital, Capital Medical University, Beijing, 101101 China

**Keywords:** Bone marrow mesenchymal stem cells (BMSCs), Schwann cells (SCs), Exosomes, Induction, RNA sequencing

## Abstract

**Background:**

Bone marrow mesenchymal stem cells (BMSCs) can differentiate into Schwann cells (SCs) during peripheral nerve injury; in our previous research, we showed that SC-derived exosomes (SC-exos) played a direct induction role while fibroblast-derived exosomes (Fb-exos) had no obvious induction role. The induction role of neural stem cell (NSC)-derived exosomes (NSC-exos) has also been widely confirmed. However, no studies have compared the induction effects of these three types of cells at the same time. Therefore, by investigating the effect of these three cell-derived exosomes upon the induction of BMSCs to differentiate into SCs, this study explored the role of different exosomes in promoting the differentiation of stem cells into SCs cells, and conducted a comparison between the two groups by RNA sequencing to further narrow the range of target genes and related gene pathways in order to study their related mechanisms.

**Materials and methods:**

We extracted exosomes from SCs, fibroblasts (Fb) and neural stem cells (NSC) and then investigated the ability of these exosomes to induce differentiation into BMSCs under different culture conditions. The expression levels of key proteins and gene markers were detected in induced cells by fluorescence immunoassays, western blotting and polymerase chain reaction (PCR); then, we statistically compared the relative induction effects under different conditions. Finally, we analyzed the three types of exosomes by RNA-seq to predict target genes and related gene pathways.

**Results:**

BMSCs were cultured by three media: conventional (no induction), pre-induction or pre-induction + original induction medium (ODM) with exosomes of the same cell origin under different culture conditions. When adding the three different types of exosomes separately, the overall induction of BMSCs to differentiate into SCs was significantly increased (*P* < 0.05). The induction ability was ranked as follows: pre-induction + ODM + exosome group > pre-induction + exosome group > non-induction + exosome group. Using exosomes from different cell sources under the same culture conditions, we observed the following trends under the three culture conditions: RSC96-exos group ≥ NSC-exos group > Fb-exos group. The overall ability to induce BMSCs into SCs was significantly greater in the RSC96-exos group and the NSC-exos group. Although there was no significant difference in induction efficiency when comparing these two groups, the overall induction ability of the RSC96-exos group was slightly higher than that of the NSC-exos group. By combining the differentiation induction results with the RNA-seq data, the three types of exosomes were divided into three comparative groups: RSC vs. NSC, RSC vs. Fb and NSC vs. Fb. We identified 203 differentially expressed mRNA target genes in these three groups. Two differentially expressed genes were upregulated simultaneously, namely riboflavin kinase (*RFK, ENSRNOG00000022273*) and ribosomal RNA processing 36 (*Rrp36, ENSRNOG00000017836*). We did not identify any co-upregulated target genes for the miRNAs, but did identify one target gene of the lncRNAs, namely *ENSRNOG00000065005*. Analysis identified 90 GO terms related to nerves and axons in the mRNAs; in addition, KEGG enrichment and GASA analysis identified 13 common differential expression pathways in the three groups.

**Conclusions:**

Our analysis found that pre-induction + ODM + RSC96/NSC-exos culture conditions were most conducive with regards to induction and differentiation. RSC96-exos and NSC-exos exhibited significantly greater differentiation efficiency of BMSCs into SCs. Although there was no statistical difference, the data indicated a trend for RSC96-exos to be advantageous We identified 203 differentially expressed mRNAs between the three groups and two differentially expressed target mRNAs were upregulated, namely riboflavin kinase (*RFK, ENSRNOG00000022273*) and ribosomal RNA processing 36 (*Rrp36, ENSRNOG00000017836*). 90 GO terms were related to nerves and axons. Finally, we identified 13 common differentially expressed pathways across our three types of exosomes. It is hoped that the efficiency of BMSCs induction differentiation into SCs can be improved, bringing hope to patients and more options for clinical treatment.

## Introduction

Over recent years, the rate of peripheral nerve injury has been increasing annually and now accounts for 2.8% of all trauma patients; furthermore, more than 50% of all patients incurring peripheral nerve injury fail to recover normal motor and sensory functionality after treatment [1, 2]. Unlike nerves in the central nervous system, peripheral nerves are able to regenerate [3]. The main clinical treatments for spinal cord injury currently include medication, surgical decompression, and rehabilitation therapy, although these methods have not achieved satisfactory treatment results. Therefore, there is an urgent need to identify methods to promote the repair and regeneration of injured peripheral nerves after trauma as this could lead to a significant improvement in a patient’s quality of life.

Cell therapy is currently considered a reliable and promising approach in the field of nerve repair, especially the transplantation of bone marrow mesenchymal stem cells (BMSCs). The common consensus is that there are two induction mechanisms involved in the induction of nerve repair following transplantation. Firstly, transplanted mesenchymal stem cells (MSCs) have been shown to secrete key neurotrophic factors, such as brain-derived neurotrophic factor (BDNF) and nerve growth factor (NGF), which then promote nerve repair [4, 5]. Secondly, transplanted MSCs can be stimulated to differentiate into SCs in the nerve injury area in vivo [6, 7]. SCs that proliferate and migrate quickly form solid cell cords (Bungner’s band) at the axon, which are nerve regeneration channels that connect the defective area and play an induction role, such that axons can continue to grow across the injured area [6, 7]. At present, the translation of SCs into clinical practice is constrained by a variety of SC sampling problems, exosome induction efficiency, and methodological issues relating to extraction and purification. Consequently, the development of new methods to induce MSCs to differentiate into SCs requires urgent investigation; this represented an important goal of this experimental study.

Exosomes are small cell-derived vesicles that are commonly found in human body fluids, including blood, urine and tissue fluids. These serve different functions in the body, including participating in intercellular signaling transduction, cell waste secretion and cell fusion [54, 55]. A number of previous studies have confirmed that SCs-derived exosomes play an important role in nerve repair and nerve regeneration after nerve injury [8–12]. Other studies have found that the transplantation of fetal neural stem cells (NSCs) into peripheral nerves may be beneficial for the treatment of muscle atrophy following peripheral nerve injury [13]. The transplantation of NSCs into damaged peripheral nerves can also lead to the differentiation of NSCs into neurons and Schwann-like cells that can secrete key neurotrophic factors and promote angiogenesis, nerve growth and myelination [14]. In our previous studies, we found that RSC96-derived exosomes(RSC96-exos) may be directly involved in the differentiation of BMSCs to SCs [15]. There are various cell components in the microenvironment surrounding a broken nerve. At present, we do not know whether other cell-derived exosomes could also directly participate in or enhance the differentiation of stem cells to SCs when co-cultured with BMSCs. Furthermore, researchers have yet to identify the precise mechanisms involved in such induction, or define the optimal induction conditions.

Therefore, In order to compare the induction effect of different exosomes and further narrow the research scope of RNA sequencing, we tested the ability of RSC96-exos, Fb derived exosomes(Fb-exos) and NSC derived exosomes (NSC-exos) to induce BMSCs to differentiate into SCs under different conditions and quantified the expression levels of Schwann cell surface markers *GFAP*, *S-100* and *P75* [16, 17]. In order to identify the potential mechanisms involved, we performed RNA sequencing on the three types of extracted exosomes to identify differentially expressed genes (DEGs) or pathways. Our intention was to identify new methods that can promote the differentiation of BMSCs into SCs, such that we can facilitate the development of new treatments for the repair of peripheral nerve injury.

## Materials and methods

### Cells

Rat BMSCs, NSCs and complete medium without serum for NSCs were purchased from Guangzhou Saiye Company (Guangdong, China). RSC96, fibroblasts and appropriate media containing Fetal Bovine Serum (FBS) were purchased from Shanghai Zhongqiao Xinzhou Company (Shanghai, China). BMSCs were cultured in α-MEM Growth Medium (HyClone; GE Healthcare Life Science) supplemented with 10% FBS(Gibco; Thermo Fisher Scientific, Inc.). The third passage of cells was used for subsequent experiments. Reagents and cytokines were as follows: α-MEM medium, 10% FBS, β-mercaptoethanol, bFGF, all-trans retinoic acid (ATRA), forskolin (FSK), rat b-FGF, PDGF (plant-derived growth factor), HRG-β1 (Heregulin-β1). We also used an Exosome extraction Kit(Lifeint, Xiamen Life Technology Interconnection Co., LTD)and an mRNA Isolation Kit(System Biosciences, LLC).

### The culture of BMSCs and their differentiation into schwann cells

The cultured third-generation BMSCs were divided into 12 experimental groups; the specific culture process is shown in Table 1. Further details relating to the induction methods were published previously [17].

**Table 1:** The culture of BMSCs under different conditions

Groups	The culture of BMSCs under different conditions
Positive control group	BMSCs were cultured with growth medium for 16 days.
BMSCs + ODM	BMSCs were cultured normally in growth medium for 3 days and were pre-induced for 4 days. Cells were then re-induced with ODM for 9 days; the culture medium was changed every 3 days.
BMSCs + RSC-exos	BMSCs were cultured normally in growth medium for 7 days; then, we added 20 µg/ml of RSC-exos for 9 days of induction culture. The media was changed every 3 days, with the exosomes being replenished each time; this strategy was applied to all groups.
BMSCs + Fb-exos	BMSCs were cultured normally in growth medium for 7 days; then, we added 20 µg/ml of Fb-exos for 9 days of induction culture.
BMSCs + NSC-exos	BMSCs were cultured normally in growth medium for 7 days; then, we added 20 µg/ml of NSC-exos for 9 days of induction culture.
BMSCs + ODM + RSC-exos	BSMCs were cultured in growth medium for 3 days and 4 days of pre-induction; then, the ODM containing 20 µg/ml of RSC-exos was replaced and re-induction culture was performed for 9 days.
BMSCs + ODM + Fb-exos	BSMCs were cultured in growth medium for 3 days and 4 days of pre-induction; then, the ODM containing 20 µg/ml of Fb-exos was replaced and re-induction culture was performed for 9 days.
BMSCs + ODM + NSC-exos	BSMCs were cultured in growth medium for 3 days and 4 days of pre-induction; then, the ODM containing 20 µg/ml of NSC-exos was replaced and re-induction culture was performed for 9 days.
induced BMSCs + RSC-exos	BMSCs were cultured in growth medium for 3 days and for 4 days of pre-induction. Then, the medium was replaced with containing 20 µg/ml of RSC-exos for 9 days.
induced BMSCs + Fb-exos	BMSCs were cultured in growth medium for 3 days and for 4 days of pre-induction. Then, the medium was replaced with containing 20 µg/ml of Fb-exos for 9 days.
induced BMSCs + RSC-exos	BMSCs were cultured in growth medium for 3 days and for 4 days of pre-induction. Then, the medium was replaced with containing 20 µg/ml of RSC-exos for 9 days.
Negative control group	RSC96 were cultured with growth medium for 16 days.

Growth medium α-MEM containing 10% FBS Pre-induction incubated with α-MEM medium containing 1 mM β-mercaptoethanol (β-ME) for 24 h before being incubated with 10% FBS and 35 ng/mL all-trans retinoic acid (ATRA) for 3 days Original induction medium(ODM) α-MEM medium consisting of 10%FBS,5µM forskolin (FSK), 10 ng/mL rat b-FGF, 5 ng/mL PDGF, 200 ng/mLHRG-β1

### Extraction, identification and quantification of exosomes

Exosomes were extracted from RSC96, Fb and NSCs using an ExoQuick-TC kit (System Biosciences, LLC). Twenty-four hours before the acquisition of RSC96s and Fbs cell supernatant, the complete medium containing FBS was replaced with the base medium without FBS. The culture medium containing BMSCs was first centrifuged (3,000 x g; 15 min; 4℃) to remove apoptotic cells and cell debris. A total of 3.3 ml of exosome precipitation solution was then added to every 10 ml of culture supernatant and incubated at 4℃ overnight. Next, the mixture was centrifuged (10,000 x g; 30 min; 4 °C), and the supernatant was discarded. The isolated exosomes were then resuspended in PBS. A BCA kit was used for quantification; the standard was diluted to different concentrations (0, 0.2, 0.4, 0.6, 0.8, and 1.0 µg/µl) and the sample and standards were loaded separately and incubated with the working solution for 30 min at 37℃ in the dark. Optical density (OD) values were then measured with a microplate reader, and sample concentrations were determined from the resulting standard curves. The amount of culture medium and exosomes required was calculated to a final concentration of 20 µg/ml and used in subsequent experiments. CD63, CD81 and calnexin were detected by Western blotting to identify the isolated exosomes.

### Transmission Electron Microscopy (TEM) of exosomes

For TEM, a total volume of 50 µl of exosomes was plated onto red wax. Then, polyvinyl acetate/carbon‑coated copper mesh was placed in the droplets and allowed to stand at room temperature for 20 min. The copper mesh was then fixed with 2% paraformaldehyde for 2 min at room temperature, washed three times with double-distilled H_2_O, and counterstained with 2% phosphotungstic acid (XiYa Reagent; Shandong West Asia Chemical Co., LTD) for 1 min at room temperature. Filter paper was then used to remove excess liquid from the copper mesh, which was subsequently dried overnight at room temperature. The extracted exosomes were then observed by TEM (magnification, x25,000).

### Nanoparticle Tracking Analysis (NTA)

Next, the exosomes were characterized by NTA (Particle Metrix, Germany). The sample pool was first cleaned with deionized water. The instrument was then calibrated with polystyrene microspheres (110 nm). Then, the sample pool was washed with 1X PBS buffer (Biological Industries, Israel). Then, exosomes derived from RSC96, Fb and NSC cells were diluted 2000-fold, 1000-fold and 500-fold with 1X PBS buffer, respectively, and then tested by injection into the NTA analyzer.

### Reverse transcriptionquantitative polymerase chain reaction (RTqPCR)

Total RNA was extracted from BMSCs using TRIzol reagent (cat. no. R0016; Beyotime Institute of Biotechnology). Total RNA was then reverse-transcribed into cDNA using a TranScript First-Strand cDNA Synthesis Kit (Beijing Exogenous Biotechnology Co., Ltd.). The conditions for the RT reaction were as follows: 25 °C for 10 min, 42 °C for 30 min and 85 °C for 5 min. Quantitative PCR was performed with SYBR GREEN Real-time Polymerase Chain Reaction Master Mix (Toyota Life Sciences; QPK-201) and an ABI PRISM 7500 Systems PCR system (Applied BioSystems; Thermo Fisher Science, Inc.). The primer pairs for each marker gene are listed in Table 2. Quantitative PCR was performed using the following thermal cycling conditions: an initial denaturing step at 50 °C for 2 min, followed by 94 °C for 2 min; then 40 cycles at 94 °C for 5 s (denaturing), annealing at 60 °C for 30 s, and a final extension step at 72 °C for 5 min. Expression levels were then calculated by the 2^−ΔΔCt^ method (57).

**Table 2:** Primer sequences for reverse transcription-quantitative PCR

Gene	Primer sequence (5’→3’)
*S100*	F: GGTTGCCCTCATTGATGTCTTCC
	R: ACCACTTCCTGCTCTTTGATTTCC
*GFAP*	F: CAAGAAACAGAAGAGTGGTATCGGT
	R: ACTCAAGGTCGCAGGTCAAGG
*NGFR/P75*	F: CCTGCTGCTGCTGCTGATTC
	R: GTTCACACACGGTCTGGTTGG
*GAPDH*	F: TCAAGAAGGTGGTGAAGCAGG
	R: TCAAGAAGGTGGTGAAGCAGG

*S100* neurospecific protein; *GFAP* glial fibrillary acidic protein; *NGFR* low affinity nerve growth factor receptor; F forward; R reverse

### Western blotting

Total protein was lysed from cells with lysis buffer (Beijing Solar Biotechnology Co., Ltd.). Then, the lysates were collected with a cell scraper. The lysates were then centrifuged (13,523 x g; 5 min., 4 °C) and the supernatant was collected and transferred to a 0.5-ml centrifuge tube. Protein samples were then mixed with 5X loading buffer, incubated at 95 °C for 5 min, rapidly cooled on ice, and stored at -80 °C until required for further experiments. Total protein was quantified using a BCA Kit; then, 40 µg of protein sample and marker (cat. SM1811; Fermentas; Thermo Fisher Scientific, Inc.) and separated via 12% sodium dodecyl sulfate – polyacrylamide gel electrophoresis(SDS-PAGE). The separated proteins were then transferred onto nitrocellulose membranes (Merck KGaA) and diluted with 5% skimmed milk in TBS-Tween-20 (TBST) for 2 h at room temperature. The membranes were then incubated overnight at 4 °C with the following primary antibodies: anti-Sox10 (1:1,000; cat. no. DF8009; Affinity Biosciences), anti-early growth response 2 (EGR2; 1:500; cat. AF0480; Affinity Biosciences), anti-S100 (1:1,000; Cat. No. AF0251; Affinity Biosciences), anti-glial fibrillary protein (GFAP; 1:1,000; cat. no. AF6166; Affinity Biosciences), and anti-low-affinity nerve growth factor receptor (NGFR; 1:2,000; cat. OM267104; Omnimabs). The following morning, the membranes were washed three times (10 min each) with 0.1% TBST at room temperature. Then, the membranes were conjugated with a goat antirabbit secondary antibody (1:5000; No. BA1054; Boster Biological Technology) and incubated on a shaker for 2 h at 37 °C. The membrane was thoroughly washed three times (10 min each) with 0.05% TBST. Protein bands were then visualized using an ECL kit (CWBio) according to the manufacturer’s protocol. Finally, we used a ChemiDo MP imaging system (Bio-Rad Laboratories) to capture representative images of the western blots.

### Immunofluorescence assays

BMSCs (2 × 10^4^ cells/well) were seeded into 24-well plates. The cells were divided into 12 groups, cultured with different media (as described earlier) and collected at pre-determined timepoints. Following the removal of the culture media, cells were washed twice with PBS, and fixed with 4% paraformaldehyde for 15 min at room temperature. Then, the cells were washed three times with PBS and blocked with 1% BSA (CWBio) at 37℃ for 30 min. Cells were then incubated with primary antibodies (NGFR/P75, Omnimabs, OM267104, 1:50; S100, Affinity, AF0251, 1:100; GFAP, Affinity, AF6166, 1:100) at 4℃ overnight, and then washed three times with PBS (5 min per wash). The next morning, cells were incubated with a secondary antibody at 37℃ for 30 min, and then washed three times with PBS 5 min per wash). DAPI was then dropped onto the cells which were then incubated in the dark for 5 min. Once the nuclei had been stained, the excess DAPI was washed off with PBS. Slides were then sealed with an anti-fluorescence quenching solution (cat.no.0100-01, Southern Biotech). Finally, images were observed under a fluorescence microscope (Olympus, BX53) and the positive rate of staining was calculated. Five fields were randomly selected for each slice to determine the total number of cells and the positive expression rate. Nuclei stained by DAPI appeared blue under UV excitation, and positive expression was red.

### RNA-sequencing

According to the manufacturer’s protocol (Lifeint, Xiamen Life Technology Interconnection Co., LTD), RNAs of RSC-exos、Fb-exos and NSC-exos were isolated using an mRNA Isolation Kit for sequencing analysis. Total RNA quantification was performed using a Nanodrop2000 (Thermo Fisher Scientific, Inc., USA) and RNA integrity was determined by an Agilent 2100 bioanalyzer (AgilentTechnology, USA). Standard cDNA libraries were then constructed using a Lifeint Transpose DNA Library Prep Kit (Lifeint, Xiamen Life Technology Interconnection Co., LTD) for Illumina and sequenced using the Illumina NovaSeq6000 platform. We used cutadapt (version 4.3) to remove the splitter sequences and filter out low-quality reads. The reads obtained from the filtered samples were then compared with the reference sequence (*Rattus norvegicus*; mRatBN7.2) by STAR (version 2.7.10b) software. The ReadCount data were derived from quantitative analysis performed by featureCounts (version 2.0.4) software; edgeR (version 3.40.2) software was used for analysis. The screening threshold for differential RNA target genes was set as a p value < 0.05 and |log2(FoldChange)| > 1.

### RNA target prediction and GO and KEGG enrichment analyses

The MiRanda database (http://www.microrna.org/microrna/home.do) was used to predict the selected target genes of differentially expressed RNAs. Gene Ontology (GO; http://geneontology.org) and Kyoto Encyclopedia of Genes and Genomes (KEGG; https://www.genome.jp/kegg/) path analysis was used to identify the target genes of RNAs. The hypergeometric distribution test was used to identify significantly enriched gene sets. *p* < 0.05 was considered statistically significant. MiRNA-mRNA interactions were selected to generate a network map using Cytoscape software (http://www.cytoscape.org/).

### Statistical analysis

SPSS version 22.0 and GraphPrism software version 9.0 were used for statistical analysis. The data was presented as the mean ± SD. All experiments were repeated three times, and all data represent the average of three independent experiments. Image-Pro Plus version 6.0 software was used to quantify the results of fluorescence immunoassays and western blotting. The results of RT-PCR, western blotting and immunofluorescence assays were analyzed by one-way analysis of variance (ANOVA) to determine whether there were differences between different groups. Tukey’s analysis was used for multiple comparisons, and a bar chart was generated. *p* < 0.05 was considered statistically significant.

## Results

### Exosome extraction, identification and electron microscopy

**Fig. 1:**
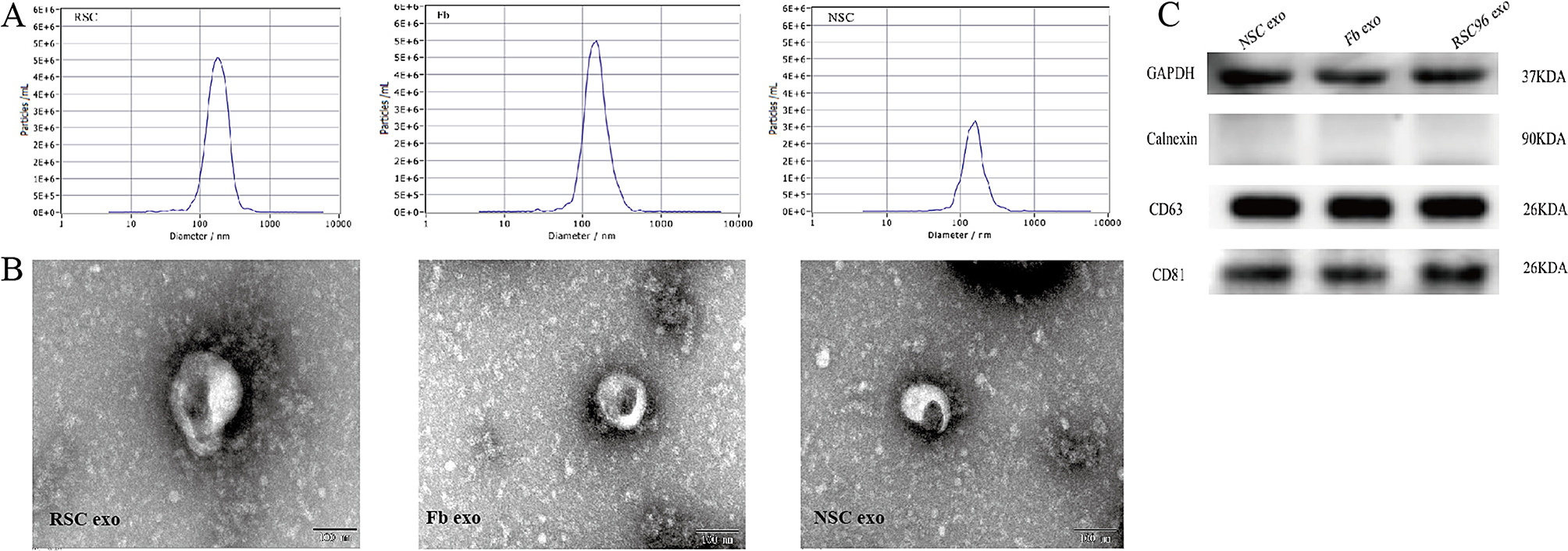
Extraction of exosomes from RSC96 cells, fibroblasts and neural stem cells. (**A**) Nanoparticle tracking analysis of the concentration and size distribution of isolated RSC96-exo, Fb-exo and NSC-exo. (**B**) Morphology of RSC96-exos, Fb-exos and NSC-exos, as observed by transmission electron microscopy. Scale bar = 100 nm. (**C**) Protein expression of the exosomal marker proteins CD81 and CD63 and the endoplasmic reticulum marker calnexin, as determined by Western blotting

Exosomes extracted from the supernatant of SCs, Fbs and NSCs (Fig. 1B) were observed using a CX41 light microscope (magnification, x200; Olympus Corporation). Exosomes appeared as irregular discoid vesicles with a diameter of approximately 50–80 nm. In addition, the mean diameters of RSC96, Fb and NSC exosomes, as determined by NTA, were 185.8 ± 2.9 nm, 147 ± 2.1 nm and 152.2 ± 1.9 nm, respectively (Fig. 1A). Exosomes were lysed, and protein quantification was performed using a BCA kit. According to a standard curve, the concentration of exosomal protein extracted from the RSC96 cell supernatant was 1.22 µg/µl while that of the Fb cell supernatant was 1.10 µg/µl; in comparison, the concentration of exosomal protein extracted from the NSC cell supernatant was 1.18 µg/µl. The extracted proteins and exosomal marker proteins (CD81 and CD63) were detected by western blotting; however, calnexin, an endoplasmic reticulum marker protein, could not be detected, thus confirming that this method was successfully able to extract exosomes (Fig. 1C).

### mRNA and protein expression levels of Schwann cell markers in BMSCs

**Fig. 2:**
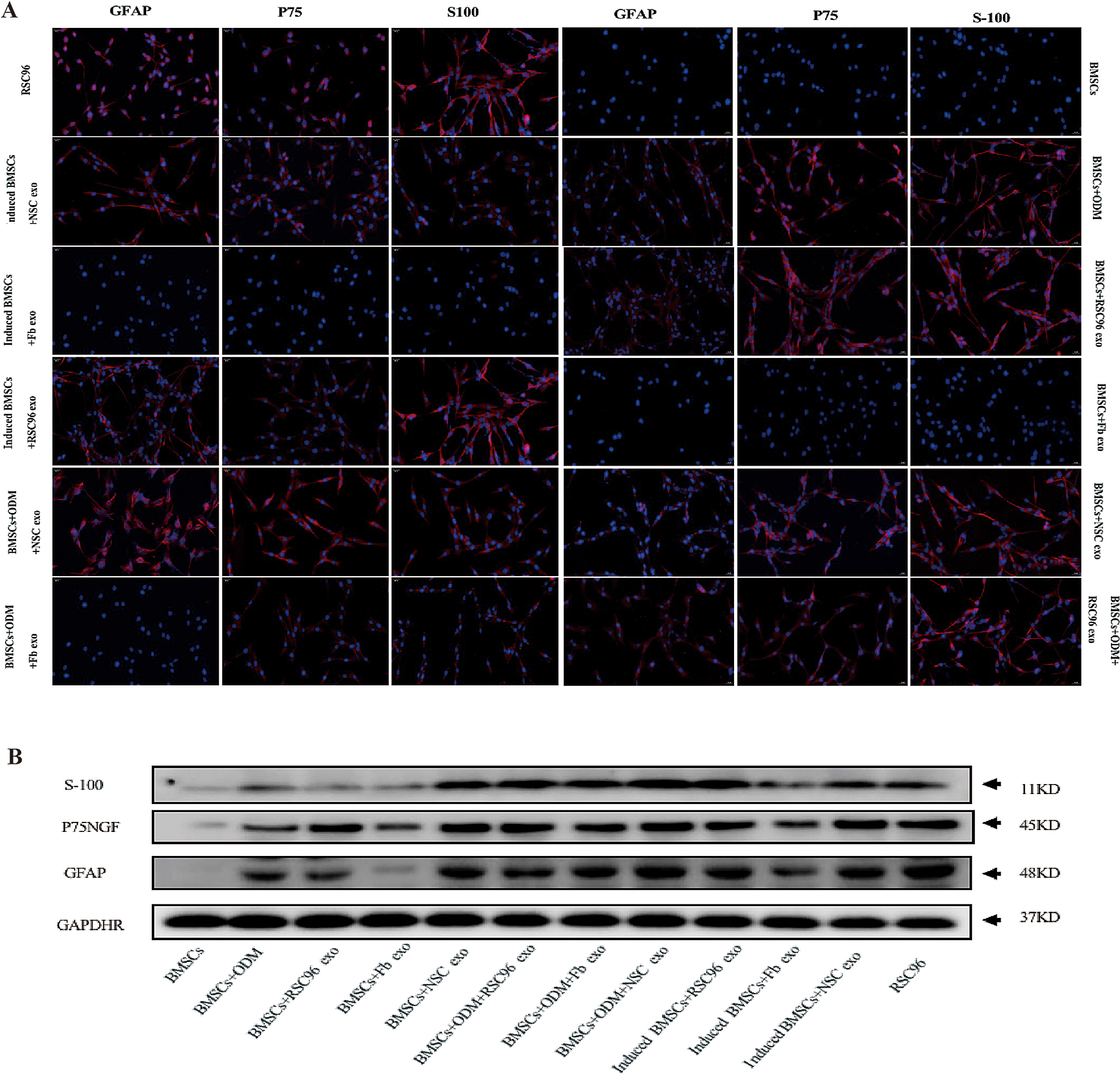
Protein expression of Schwann cell markers in differentiallyinduced BMSCs(*n* = **3)**. (**A**) Representative immunofluorescence micrographs showing the expression levels of Schwann cell markers (*S100*, *GFAP*, *P75NGRF*) in twelve experimental groups (BMSCs, BMSCs + ODM, BMSCs + RSC96-exos, BMSCs + Fb-exos, BMSCs + NSC-exos, BMSCs + ODM + RSC96-exos, BMSCs + ODM + Fb-exos, BMSCs + ODM + NSC-exos, induced BMSCs + RSC96-exos, induced BMSCs + Fb-exo, induced BMSCs + NSC-exo and RSC96) (magnification, x100). Scale bar, 50 μm. (**B**) Representative western blotting of the protein expression levels of *S100*, *GFAP*, and *P75NGRF* in the twelve groups

Immunofluorescence results (Fig. 2A) showed that the positive expression levels of *S100*, *GFAP*, *NGFR* and mRNA of rat BMSCs increased to different degrees after the differentiation of BMSCs under different culture conditions and when treated with different exosomes. BMSCs exhibited the best induced differentiation effect during pre-induction and ODM culture. Compared with NSC-exos and Fb-exos, the addition of RSC96-exo improved the differentiation efficiency of BMSCs to SCs. These results were similar to those provided by western blotting (Fig. 2B).

### Immunofluorescence, Western blotting, mRNA and protein expression

**Fig. 3:**
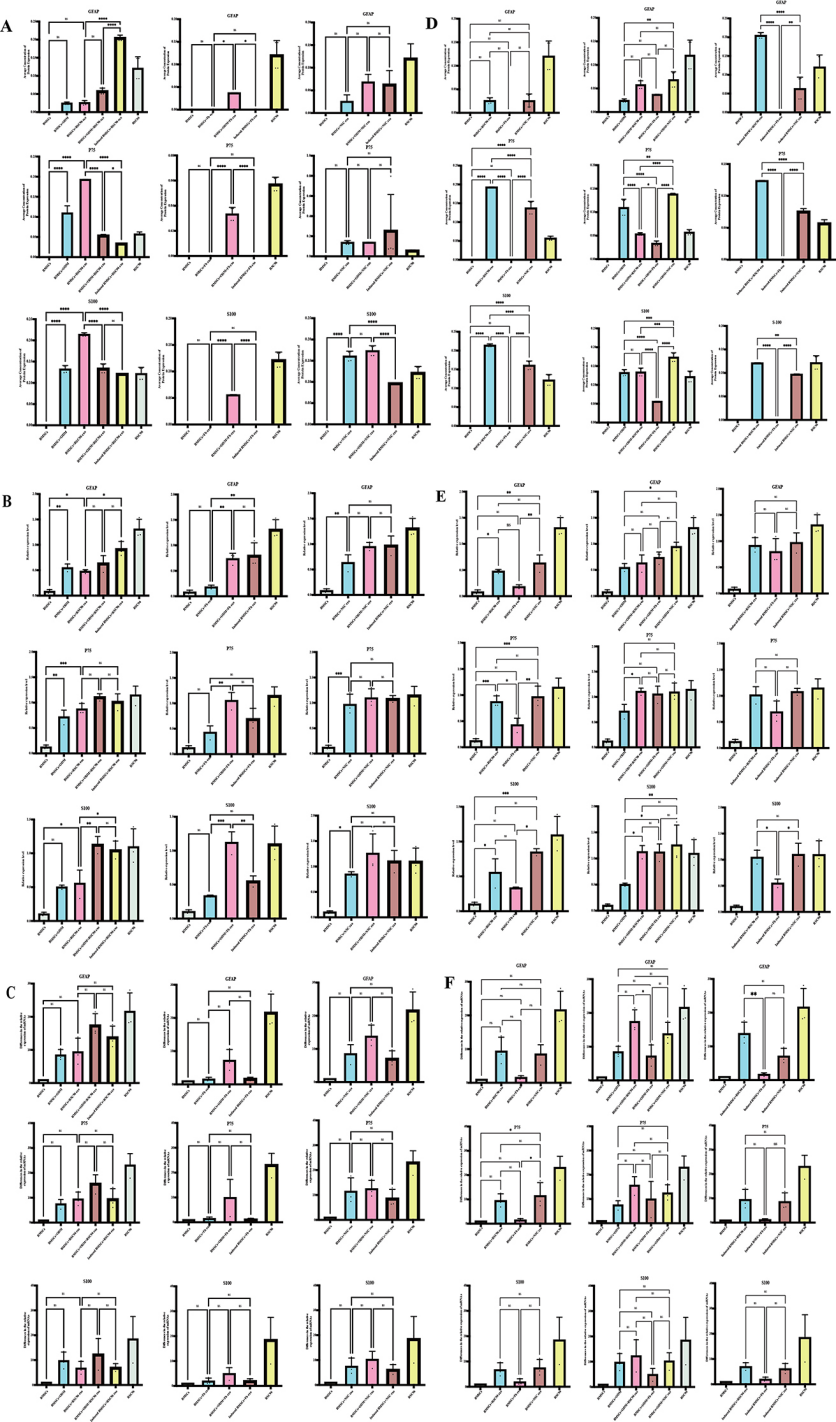
MRNA and protein expression levels of Schwann cell markers in differentiallyinduced BMSCs(*n* = **3)**. (**A**-**C**) Induction effect of exosomes (RSC96-exos, Fb-exos and NSC-exos) on BMSCs under different culture conditions. The induction effect was evaluated by quantifying the expression levels of Schwann cell markers (*S100*, *GFAP*, *P75NGRF*) by fluorescence immunoassays, western blotting and PCR. (**D**-**F**) Induction of exosomes (RSC96-exos, Fb-exos and NSC-exos) on BMSCs under the same culture conditions. The induction effect was evaluated by quantifying the expression levels of Schwann markers (*S100*, *GFAP*, *P75NGRF*) by fluorescence immunoassays, western blotting and PCR. Data are presented as the mean ± SD; *P < 0.05, **P < 0.01, ***P < 0.001 and ****P < 0.0001

BMSCs were induced to differentiate under different culture conditions and the expression levels of Schwann cell surface markers (*S100*, *GFAP*, and *P75NGFR*) and total RNA extracted from the BMSCs were analyzed by fluorescence immunoassay, western blotting and PCR; our analysis found that the expression levels of these markers varied according to different culture conditions (Fig. 3). We analyzed the induction and differentiation effects of BMSCs under three different culture conditions (Fig. 3A-C). The protein and mRNA expression levels of *S100*, *GFAP*, *P75NGFR* of the SCs after the addition of 3 exosomes were analyzed, respectively; expression levels were highest in pre-induced + ODM + exosome group; this was followed by the pre-induced + exosome group, and then the non-induced + exosome group. Under the same culture conditions, we added RSC96-exos, NSC-exos and Fb-exos; then, we analyzed the effects of these additions on the induced differentiation of BMSCs into SCs (Fig. 3D-F). Analysis showed that the overall protein and mRNA expression of *S100*, *GFAP*, *NGFR* increased significantly when adding these RSC96-exos, NSC-exos. However, compared with NSC-exos, the induction ability of RSC96- exos was higher or lower, some had statistical significance, and some had no statistical significance. Overall, the analysis showed that the induction ability of the two groups (RSC96-exos and NSC-exos) was very similar. However, the induction ability of RSC96-exos was slightly stronger than that of NSC exos, although this was no statistical significance. Following treatment with Fb-exos, the protein and mRNA expression levels of *S100*, *GFAP* and *P75* in BMSCs were slightly increased, but significantly lower than those in the other two experimental groups, as follows: RSC96-exos ≥ NSC-exos > Fb-exos.

To summarize, BMSCs were cultured with exosomes of the same cell origin under different culture conditions with conventional (no induction), pre-induction, or pre-induction + original induction medium (ODM). Analysis after adding three types of exosomes showed: pre-induction + ODM + exosome group > pre-induction + exosome group > non-induction + exosome group. When using exosomes from different cell origins under the same culture conditions, the induction of BMSCs into SCs was highest in the RSC96-exos group, followed by the NSC-exos group and the Fb-exos group; the overall induction effect was significantly higher in the RSC96-exos and NSC-exos group. Although there was no statistical difference in induction efficiency between these two groups, the overall induction ability of the RSC96-exos group was slightly higher than that of the NSC-exos group.

### RNA expression profiles of NSCs, RSCs and fbs

**Fig. 4:**
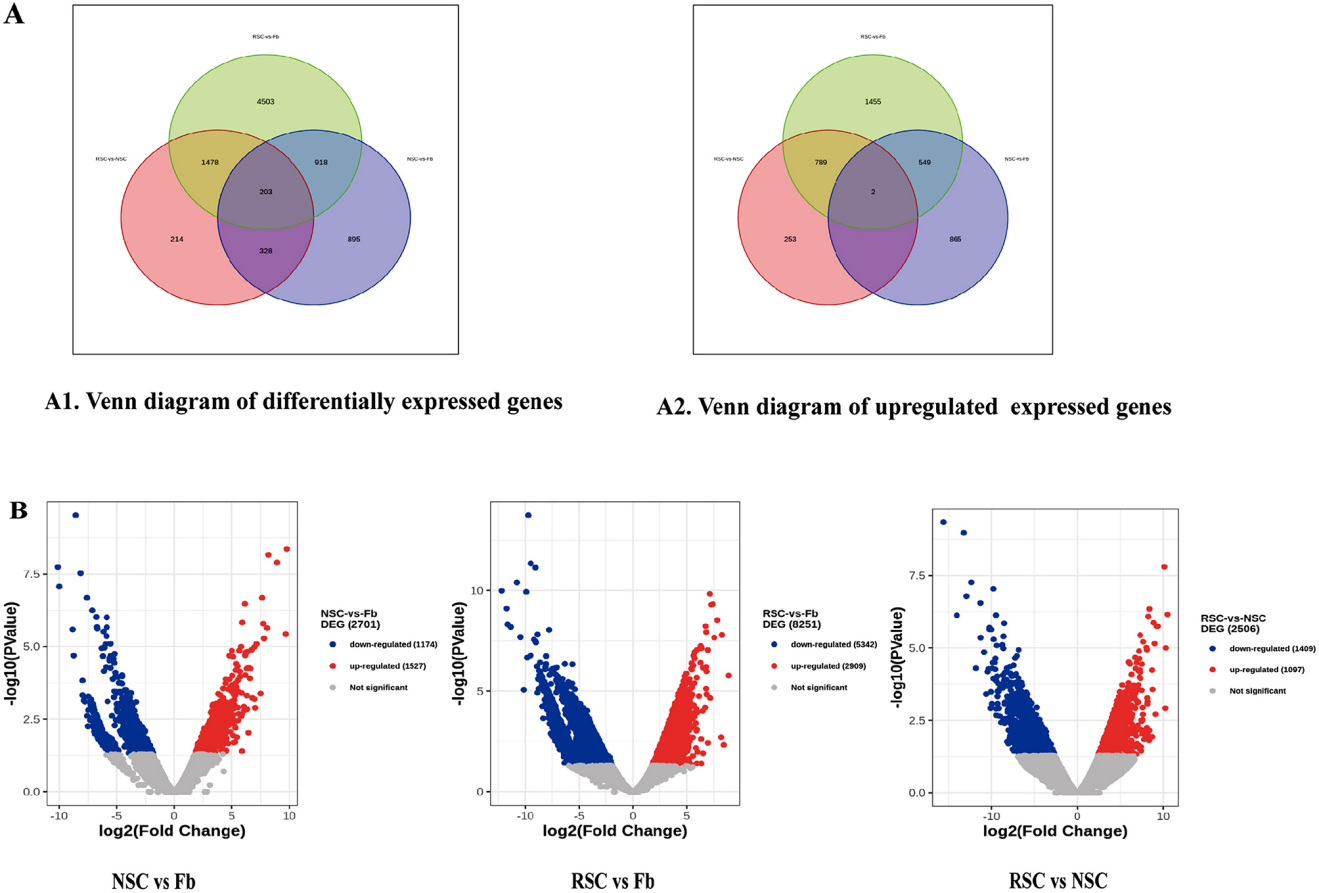
The mRNA expression profile of three experimental groups: NSC vs. Fb, RSC vs. Fb, and NSCvs RSC. (**A**) The number of differentially expressed mRNAs (up- or down-regulation) in the form of a Venn diagram. (**B**) Volcano map showing the distribution of differentially expressed mRNA target genes

Next, RNA-sequencing was used to predict the RNA levels of the differentially expressed genes in the three types of exosomes: NSC-exos, RSC-exos and Fb-exos. Fb-exos had almost no induction effect on BMSCs differentiation into SCs cells, while NSC-exos and RSC-exos had obvious induction effects. Therefore, we compared RNA from NSC-exos and RSC-exos with Fb-exos respectively, excluded non-inducible genes, and further narrowed the scope of target gene research. In order to further narrow the research scope of the RNA target genes, sequencing was combined with the induction ability of exosomes; the induction efficiency was highest in the RSC96-exos, followed by the NSC-exos group and then the Fb-exos group. We compared RNA levels between paired groups of exosomes from the three groups. Regardless of whether gene expression was up- or down-regulated, we identified groups showing statistical significance (*P* ≤ 0.05); for our analysis, we created three paired groups: NSC vs. Fb, RSC vs. Fb, and NSC vs. RSC. When analyzing these groups, we found no co-upregulated target genes of miRNAs. We only identified one lncRNA, namely ENSRNOG00000065005; however, this lncRNA was novel and could not be identified by gene websites or by the literature. Thus, we focused on mRNA target genes. A total of 8539 differentially expressed genes (DEGs) of mRNA were identified by RNA-sequencing, including the NSC vs. Fb, RSC vs. Fb and the NSC vs. RSC. Groups. In the NSC vs. Fb comparison, there were 1416 up-regulated DEGs and 928 down-regulated DEGs; in the RSC vs. Fb comparison, there were 2795 up-regulated DEGs and 4307 down-regulated DEGs; in the NSC vs. RSC comparison, there were 1044 upregulated DEGs and 1179 down-regulated DEGs. In total, 203 DEGs were common to the three groups (Fig. 4A1). Further analysis showed that two upregulated DEGs were common to all three groups (Fig. 4A2), namely riboflavin kinase (RFK, ENSRNOG00000022273) and ribosomal RNA processing 36 (Rrp36, ENSRNOG00000017836) in the future. Figure 4B shows a volcano map depicting the distribution of DEGs across the three groups.

#### GO, KEGG and GASA analysis

**Fig. 5:**
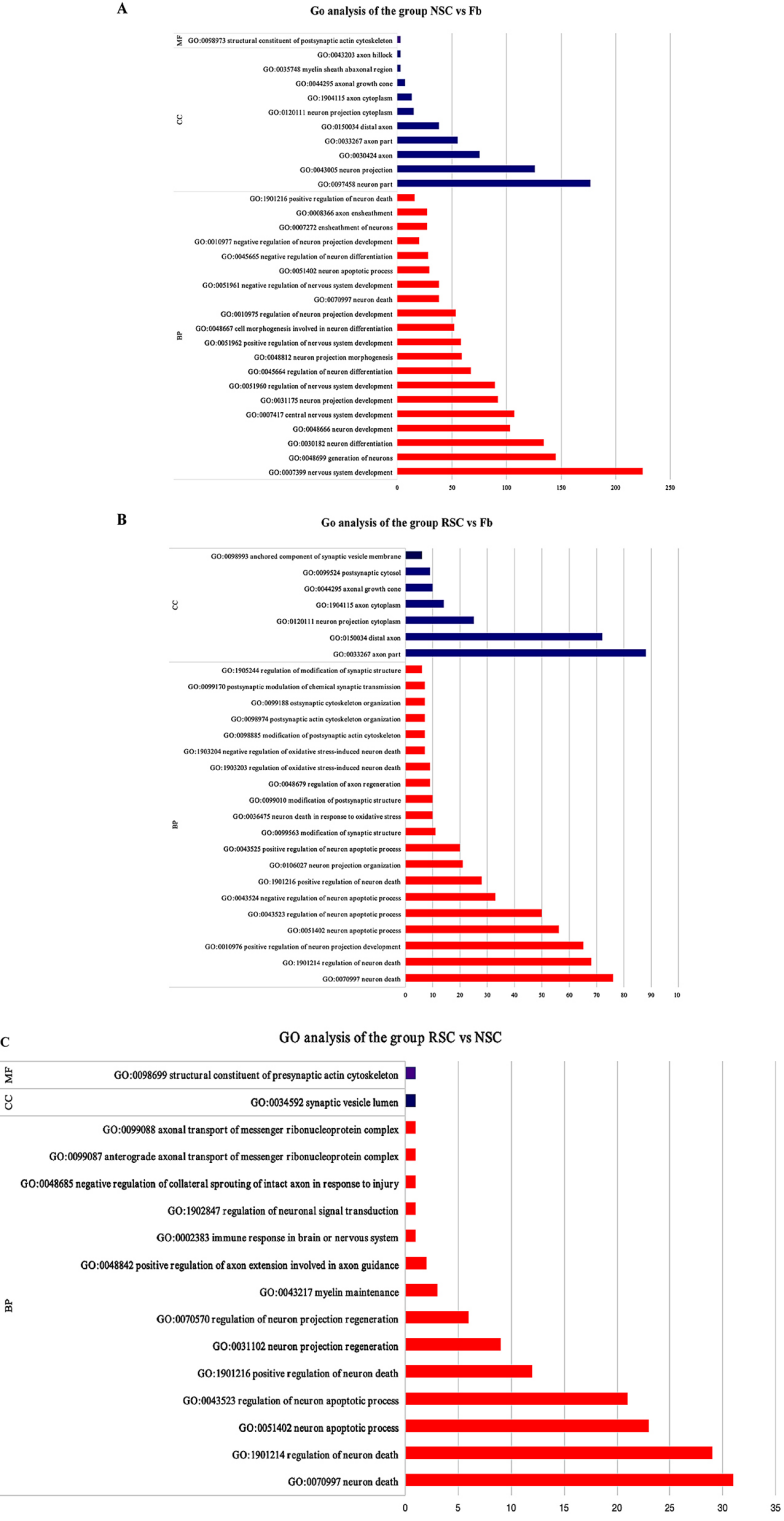
Significant GO terms and pathways for selected target genes. We selected the top 20 GO terms with the most significant enrichment for visualization; if there were less than 20 GO terms, all terms are displayed. (**A**) GO terms related to neurons, axons, myelin and nerves are listed for NSC vs. Fb; (**B**) GO terms related to neurons, axons, myelin and the nervous system are listed for RSC vs. Fb; (**C**) GO terms related to neurons, axons, myelin and nerves are listed for RSC vs. NSC. The red column represents the list of hits for biological processes, the blue column represents the list of hits for cellular components, and the purple column represents the list of hits for molecular function

The number and distribution of DEGs associated with GO terms enriched in the biological process, cellular component and molecular function categories were reflected by a histogram of differentially expressed genes identified by GO enrichment analysis and also allowed us to predict the functionality of the target genes. We selected 20 GO terms with the most significant enrichment (Fig. 5B-D); if there were less than 20 GO terms, all GO terms were shown. In the analysis of molecular function, we compared the three groups with regard to the most enriched genes and found that there were three identical pathways associated with DEGs: protein dimerization activity, protein binding, and identical protein binding (Fig. 5B; Table 3). In the analysis of biological processes, we compared the most enriched genes between the three groups and found that there were no common DEGs or pathways (Fig. 5 C, Table 3). In our analysis of cell components, we compared the three groups with regard to the most enriched genes and found that there were two DEGS or pathways that were common to the three groups: the cytoplasmic component and the cytoplasm (Fig. 5D; Table 3). Our conditions for selection were (1) *P* < 0.05 and (2) GO terms related to neuron, myelin, or axonal function. A detailed comparison between the three groups is shown in Fig. 5; a total of 90 GO terms met our specific criteria, suggesting that differentiated cells may also have a role in regulating the peripheral nervous system, such as axons and neurons, which needs further verification. Our results suggest that these target genes may play an important role in the differentiation of BMSCs into SCs induced by RSC-exos or NSC-exos.

**Table 3:** Enrichment results of the same differential gene among different groups

		NSC vs. Fb/RSC vs. Fb	RSC vs. Fb/RSC vs. NSC	NSC vs. Fb/RSC vs. NSC
GO Enrichment of Genes	Biological Process	None	Cell death Negative regulation of biological process Negative regulation of cellular process	None
	Cellular component	*Cytoplasmic part* *Cytoplasm* Catalytic complex Mitochondrial part	*Cytoplasmic part* *Cytoplasm* Intracellular vesicle Cytoplasmic vesicle Vesicle Extracellular region Extracellular region part	*Cytoplasmic part* *Cytoplasm* Membrane-bounded organelle Intracellular Intracellular part Membrane-bound organelle
	Molecular Function	*Protein dimerization activity* *Protein binding* *Identical protein binding* Ubiquitin conjugating Enzyme activity	*Protein dimerization activity* *Protein binding* *Identical protein binding* Protein homodimerization activity	*Protein dimerization activity* *Protein binding* *Identical protein binding* Catalytic activity Molecular function regulator Chemokine activity binding
Intersection of differential gene enrichment analyses in KEGG and GSEA		Spliceosome, Oocyte meiosis, Cell cycle, Carbon metabolism, Glycine, serine and threonine metabolism, DNA replication, Cysteine and methionine metabolism, Dopaminergic synapse, Proteasome, Pyruvate metabolism, RNA polymerase, mRNA surveillance pathway, SNARE interactions in vesicular transport		

Regardless of whether gene expression was up- or down-regulated, we identified groups showing statistical significance (*P* ≤ 0.05); for our analysis, we created three paired groups: NSC vs. Fb, RSC vs. Fb and NSC vs. RSC. The italized sections represent three groups of identically expressed genes

**Fig. 6:**
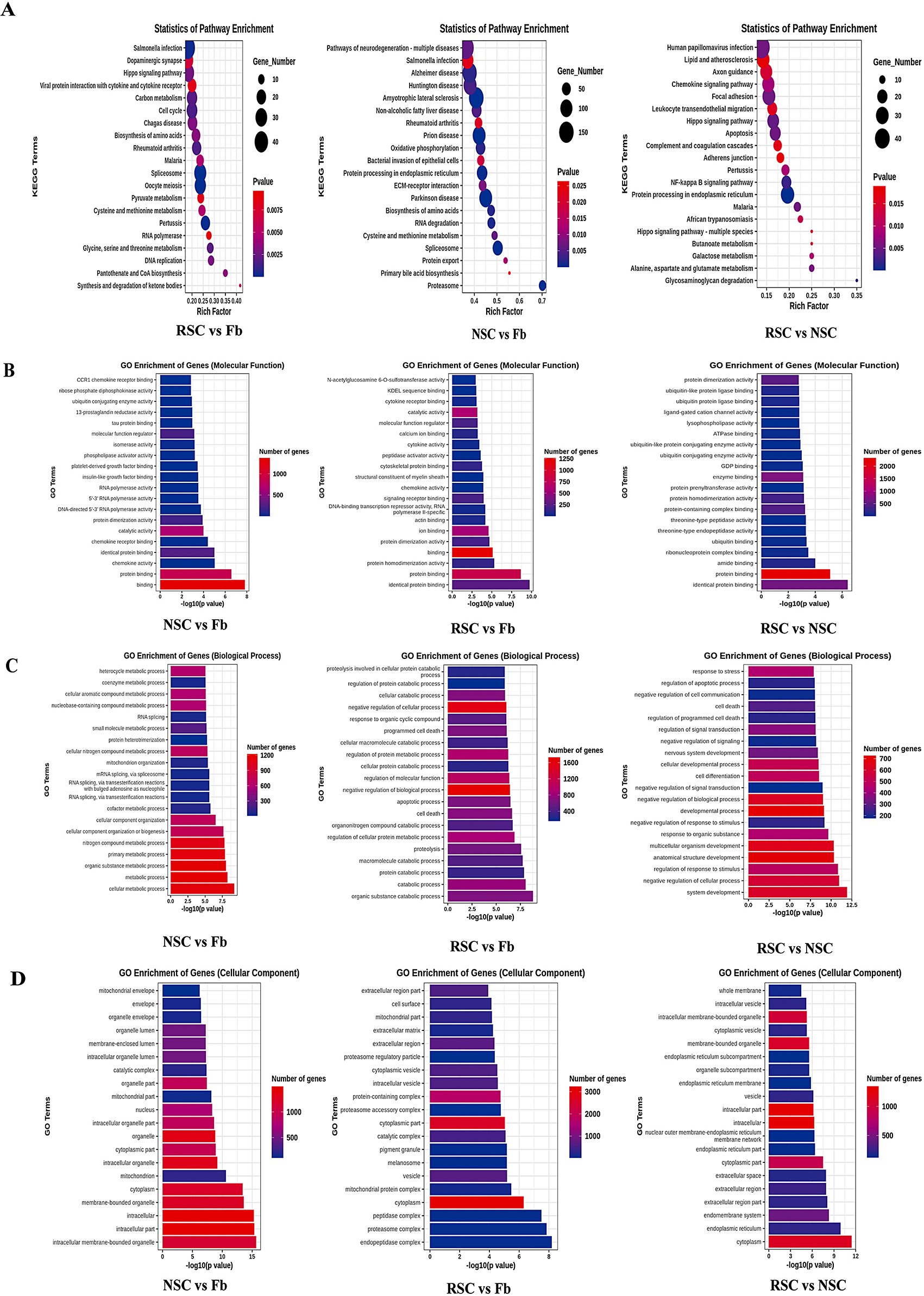
Significant GO terms and pathways of the target genes. (**A**) The results arising from KEGG enrichment analysis are displayed graphically as a scatterplot. (**B**-**D**) The number and distribution of differentially expressed genes arising from GO term analysis enriched by Molecular Function, Biological Process and Cellular Component, as depicted by histograms. This figure features 20 GO terms, with the most significance; if there were less than 20 GO terms, then all terms are shown

The results of KEGG enrichment analysis are displayed as scatter plots; in Fig. 6A; ‘Rich Factor’ refers to the ratio of the number of DEGS enriched in a pathway to the number of annotated genes. The greater the Rich Factor, the greater the degree of enrichment. We selected 20 pathway entries with the lowest P-values for visualization; if there were less than 20 enriched pathway entries, all entries were visualized (Fig. 5A). Then, we performed intersection analysis for the three groups in terms of KEGG enrichment analysis results and GASA analysis results (Table 3). Analysis showed that the common DEGs or pathways may be associated with the spliceosome; oocyte meiosis; the cell cycle; carbon metabolism; glycine, serine and threonine metabolism; DNA replication, cysteine and methionine metabolism; dopaminergic synapses; the proteasome; pyruvate metabolism; RNA polymerase; the mRNA surveillance pathway, and SNARE interactions in vesicular transport.

## Discussion

Neural repair after peripheral nerve injury involves a series of interaction mechanisms, including axonal regeneration, the activation of SCs, vascular regeneration, and the regulation of inflammation [18, 19]. The cells involved in the repair process of nerve injury predominantly include SCs, macrophages, fibroblasts, and lymphocytes [20]. Following peripheral nerve injury, the distal axons and myelin sheath, as well as some proximal axons, first expand but then collapse into fragments and droplets; this is a process referred to as Waller’s degeneration [21, 22]. SCs de-differentiate, proliferate, and form Bungner bands, which help to guide the growth of proximal nerves [23]. Previous studies have shown that the maintenance and development of peripheral nerves depends on local signal transduction between the axons and SCs [24] and that de-differentiated SCs proliferate and provide a mechanical matrix and growth factors from which to regenerate axons. To support axonal growth, axons interact with proteins on the SCs and trigger the SCs to undergo maturation and myelination processes [25].

The detailed mechanism by which exosomes induce the differentiation of BMSCs into SCs remains unclear. Exosomes are known to regulate various biological processes between cells, predominantly *via* the transfer of DNA, RNA, proteins and lipids; of these, mRNA plays a major role in gene expression and the translation of proteins. Our previous in vitro experiments confirmed that exosomes from SCs could improve the efficiency of BMSCs to differentiate into SCs. In addition, in vivo studies have confirmed that SC-exos cannot only promote axonal growth but also promote axonal regeneration after injection of exosomes when nerve compression injury [26]. However, the active components and precise mechanisms involved in this form of induction have yet to be elucidated. The exosomes of SCs are associated with complex components and a diverse range of functions; moreover, most previous studies on the exosomes of SCs focus mostly on their supporting role in nerve regeneration [8, 9]. First, although many studies have attempted to identify the exosomal components of SCs, very little is known about the precise identification of these components [27–29]. Second, the precise mechanism by which exosomes enhance the differentiation of MSCs into SCs is unknown. Multiple studies have shown that miRNAs derived from SC-exosomes (such as miRNA340 and miR-221/222) are important regulators of SC regeneration after nerve injury. Moreover, the expression of many proliferating miRNAs is positively regulated by Sox10, which is also a known participant in stem cell development, differentiation, and remyelination [30–36]. It has also been reported that a large number of miRNAs in axons or nerve endings may be directly transferred from SCs [37].

According to our current experimental data and previous literature, several mRNAs have differential effects on peripheral nerve damage or SC differentiation. KEGG and GSEA analysis identified several common pathways and DEGs, such as oocyte meiosis and the proteasome. The down-regulation of oocyte meiosis showed that the proliferation of Schwann cells decreased after bone marrow formation [38]. In the present study, we found that oocyte meiosis was enriched when the expression of SC markers increased; this finding concurred with the previous literature. We also found that proteasome expression was enriched; the ubiquitin-proteasome system (UPS), lysosomes and autophagy are protein degradation systems that play key roles in the regulation of a variety of physiological events in cells, including cell response to injury. Previous studies have shown that the inhibition of UPS activity can delay the degradation of myelin and axons after nerve injury. Appropriate regulation of the UPS is essential to maintain the normal and regenerative function of peripheral nerves; however, we know little about the precise regulatory mechanism involved [39]. This work provides a new paradigm via rationally applying SC-exos and RSC-exos as promising therapeutic options for repairing peripheral nerve injury.

NSCs have been proven to be suitable donor cells for tissue engineering research due to their potential for multidirectional differentiation, strong plasticity and migration ability, and low immunogenicity [40]. Previous studies have confirmed that NSCs can promote the repair of peripheral nerve injury in a variety of ways; for example, by secreting a variety of neurotrophic factors. Furthermore, the implantation of NSCs into the nervous system has been shown to promote axonal regeneration and form a Schwann-like peripheral myelin sheath [40–45]. NSCs can also promote repair after peripheral nerve injury by activating the Sirt-1 signaling pathway to inhibit the inflammation of activated macrophages [40–45]. NSCs can also improve the compatibility of cell regeneration and transplantation with surrounding tissues, while the low immunogenicity of NSCs may reduce or even avoid the use of immunosuppressants [46]. Therefore, NSCs and their exosomes could also be used as suitable donor cells to promote the regeneration of peripheral nerves.

Thus far, research has failed to identify a safe inducer with a strong induction effect from a non-chemical source in vivo, although the Dezawa method is a relatively well recognized in vitro induction method [17]. The pre-inducer β-ME has been shown to induce the formation of neurite-like processes [47, 48]. All-trans-retinoic acid(ATRA)is a morphological factor that plays a role during development by inducing embryonic stem (ES) cells and NSCs to differentiate into nerve cells; furthermore, ATRA has been shown to regulate various transcription factors that are critical for early neuronal decision making [49, 50]. Therefore, it can be inferred that these two mechanisms can be used as trigger factors to induce changes in the morphological and transcriptional characteristics of MSCs. In addition, basic fibroblast growth factor (bFGF), platelet-derived growth factor(PDGF) and Histidine Rich Glycoprotein (HRG) have been shown to be effective in the differentiation and proliferation of glial cells and SCs [51, 52]. HRG is a subtype of neuregulin, which is known to exert a guiding influence on the cell fate and can induce neural crest cells to selectively develop into SCs [53]. Since the induction effect of the Fb-exos was minimal, the BMSCs + ODM group was compared with the pre-induction + RSC-exos/NSC-exos group, respectively; we found that the RSC-exos and NSC-exos groups showed stronger induction effects than ODM. When BMSCs were cultured in the pre-induction + ODM condition, we found that the differentiation of BMSCs into SCs increased significantly after adding RSC-exos and NSC-exos to the medium, thus indicating that the induction effect of pre-induction + ODM + RSC96/NSC-exos was more effective. Although the ODM induction mechanism has been confirmed, whether exosomes have a similar induction effect, and whether the combined induction is better than a single induction effect, requires further investigation. At present, our research is still limited to the level of exosome induction and gene screening; future research needs to be more in-depth. In our future research, we will focus on the verification of target genes and related pathways.

A literature search revealed only a few studies related to RFK, an enzyme that catalyzes chemical reactions, catalyzes the phosphorylation of riboflavin to form flavin mononucleotide (FMN), and represents a precursor of flavine adenine dinucleotide (FAD). Riboflavin (7,8-dimethyl-10-ribityl-isoalloxa zine), also known as vitamin B2, its most important biologically active forms are FAD and FMN. The metabolic function of the flavin coenzymes is as electron carriers in a wide range of oxidation and reduction reactions essential for all metabolic processes, including the electron transport chain. They also have a role in chromatin remodelling, DNA repair, protein folding, and apoptosis [56–58]. Riboflavin has an important role in myelin formation, selectively intervening in peripheral nerve myelin synthesis [56, 57]. Riboflavin deficiency has been shown to cause severe peripheral neve demyelination in young, rapidly growing chickens. Its deficiency can cause a debilitating peripheral neuropathy, and mortality, in poultry flocks, and it can also be a useful experimental animal model to study the pathogenesis of reliably reproducible peripheral nerve demyelination [59]. Other studies have identified riboflavin as a potential neuroprotective agent that canameliorate oxidative stress, mitochondrial dysfunction, neuroinflammation, and glutamate excitotoxicity, all of which are involved in the pathogenesis of Parkinson’s disease, migraine, and other neurological disorders [60]. Therefore, RFK is known to be closely associated with nerve repair, but the specific mechanism is still unknown. In our future research, we will focus on mechanisms of action, pathways and relationship between RFK and peripheral nerve repair. There is little research on Rrp36 and almost nothing to do with neural repair, which needs further study.

## Conclusions

In this study, we found that pre-induction + ODM + RSC96/NSC-exos culture conditions were most conducive to inducing the differentiation of BMSCs to SCs. Furthermore, the addition of exosomes following pre-induction was better than without induction. Both RSC96-exos and NSC-exos significantly improved the efficiency of BMSCs to differentiate into SCs. Although there was no statistical difference, RSC96-exos showed a trend for better differentiation induction. In addition, we used bioinformatics analysis to predict the potential functions and mechanisms of differential mRNAs and their key target genes. The analysis identified 203 differentially expressed mRNAs, 2 DEGs that were upregulated, namely riboflavin kinase (*RFK, ENSRNOG00000022273*) and ribosomal RNA processing 36 (*Rrp36, ENSRNOG00000017836*), and 90 GO terms related to nerves and axons. In addition, we identified 13 common differentially expressed pathways by performing KEGG enrichment and GASA analysis. Our future research will focus on investigating the pathways and mechanisms of action of the target genes of differentially expressed mRNAs. In the future, mRNA carriers may be transplanted to improve the therapeutic effect so as to enrich the repair of peripheral nerve injury or improve the induction efficiency.

## Data Availability

No datasets were generated or analysed during the current study.
