# Correction: The effects of exosomes originating from different cell sources on the differentiation of bone marrow mesenchymal stem cells into Schwann cells

**DOI:** 10.1186/s12951-024-02604-3

**Published:** 2024-07-10

**Authors:** Xianxiang Zhang, Weiwei Zhang, Hao Sun, Hui Wang

**Affiliations:** Department of Otolaryngology and Head and Neck Surgery, Beijing Luhe Hospital, Capital Medical University, Beijing, 101101 China

**Correction: J Nanobiotechnol (2024) 22:220**

10.1186/s12951-024-02450-3

In this article, the order that the authors appeared in the author list was incorrect.

The incorrect order of authors are Hui Wang, Xianxiang Zhang, Weiwei Zhang and Hao Sun

The correct order of authors is given below

Xianxiang Zhang, Weiwei Zhang, Hao Sun, Hui Wang.

The original article has been corrected.

